# Ensiled *Pleurotus ostreatus* based spent mushroom substrate from corn: *In vitro* gas production, greenhouse gas emissions, nutrient degradation, and ruminal fermentation characteristics

**DOI:** 10.3934/microbiol.2025001

**Published:** 2024-12-12

**Authors:** Chika C. Anotaenwere, Omoanghe S. Isikhuemhen, Peter A. Dele, Michael Wuaku, Joel O. Alabi, Oludotun O. Adelusi, Deborah O. Okedoyin, Kelechi A. Ike, DeAndrea Gray, Ahmed E. Kholif, Uchenna Y. Anele

**Affiliations:** 1 Department of Animal Sciences, North Carolina Agricultural and Technical State University, Greensboro, NC 27411, USA; 2 Department of Natural Resources and Environmental Design, North Carolina Agricultural and Technical State University, Greensboro, NC 27411, USA; 3 Department of Pasture and Range Management, Federal University of Agriculture, Abeokuta, Ogun State Nigeria; 4 Dairy Science Department, National Research Centre, 33 Bohouth St. Dokki, Giza 12622, Egypt

**Keywords:** batch culture, corn silage, degradability, ensiling duration, *in vitro* fermentation, spent mushroom substrate

## Abstract

The present study evaluated varying inclusion levels (10%, 20%, 30%, 40%, and 50%) of spent mushroom substrate (SMS) derived from *Pleurotus ostreatus*, ensiled for 0, 21, 42, and 64 d, using an *in vitro* batch culture technique. The study employed a 6 × 4 factorial design with six inclusion levels and four ensiling durations. The batch culture was conducted over 24 h across two runs. Gas production (GP), greenhouse gases (GHG) production, nutrient degradability, and volatile fatty acids (VFA) were measured. Significant interactions (P < 0.01) between ensiling duration and diet were observed for the concentrations of different nutrients and GHG production. SMS levels in diets increased (P < 0.001) dry matter (DM), neutral (NDF), and acid (ADF) detergent fiber concentrations but decreased crude protein (CP) and cellulose levels. Ensiling period decreased (P < 0.001) DM, NDF, acid-detergent lignin (ADL), and hemicellulose concentrations but increased non-structural carbohydrates (P < 0.05). Diets with higher SMS levels had lower (P < 0.001) GP, methane (CH_4_), and carbon dioxide (CO_2_) production, together with increased degradability of DM, NDF, ADF, and ADL. Conversely, extending ensiling increased CH_4_ and CO_2_ production, degradability of DM, and proportions of acetate and propionate but decreased NDF and ADF degradability. Total VFA and butyrate were highest (P < 0.05) in the diet with 50% SMS inclusion. In conclusion, SMS can replace up to 50% of corn silage in the diets of beef and non-lactating dairy cows; however, extending the ensiling duration is not recommended.

## 1. Introduction

The world faces an unprecedented challenge to enhance agricultural productivity while ensuring environmental sustainability due to exponential population growth, climate change, and increasing food demand [1]. The global population is projected to reach 9.7 billion by 2050, necessitating a 60% increase in food production [2]. Despite all the improvements and progress made, current agricultural practices continue to contribute significantly to environmental degradation [3], emphasizing the urgency for a shift toward more sustainable agricultural practices.

The utilization of spent mushroom substrate (SMS), a byproduct of mushroom cultivation, is gaining attention in the feed and livestock industries [4]–[6]. In the USA, approximately 5 kg of spent mushroom substrate (SMS) is produced for every kilogram of mushrooms after the fruiting stage of mushroom cultivation [6],[7]. This is seen by mushroom producers as waste, resulting in 5 million tons of SMS solid waste being disposed of annually [7]. Ruminant feeding is one of the strategies to utilize SMS [5],[8].

A wide range of nutrients and bioactive compounds in SMS show that it could be used as a natural feed or a feed additive for beef and dairy cattle [5],[6],[9]. In addition, SMS can be obtained cheaply from mushroom-processing plants and utilized as a cost-effective ruminant feed, particularly in countries where livestock farmers depend on imported animal feeds [6],[8]. Due to its high moisture content, SMS decomposes rapidly; hence, it must be processed quickly. During the growth of mushrooms, it produces active enzymes that could boost dry matter (DM) digestibility when SMS is fed to livestock [5]. Additionally, mushrooms produce abundant active ingredients, including polysaccharides, vitamins, and trace amounts of minerals that help balance the microbiota in livestock and potentially enhance growth performance [5].

The main problem with feeding SMS is the low nutritive value due to high fiber content [5]. Ensiling is an important roughage processing method that can improve the palatability of feed, prolong the storage time of high-moisture content feed, and maintain the original nutrient level [10]. Therefore, ensiling with some additives is a strategy to improve its nutritive value before feeding. Ensiling can result in a decrease in the crude fiber and an increase in the contents of protein, fat, and ash [10]. Silage storage time also promotes changes in quality; however, the magnitude of these changes is not fully elucidated. Silages stored for extended periods could result in reduced concentrations of soluble carbohydrates, as they are used by microorganisms as a substrate during fermentation, thereby increasing the concentrations of the fibrous compounds [11]. Senger et al. [12] reported that increasing storage time modifies the fibrous structure of silage due to acid digestion that occurs during the fermentation process (beyond 21 d). In an *in vitro* experiment, Olagunju et al. [6] stated that *Pleurotus ostreatus*–treated corn stover increased CP, non-fiber carbohydrates, and ash by 58.5%, 118%, and 25.8%, respectively, compared to the untreated corn stover. Similarly, they reported a decrease in fiber concentration. Additionally, they also observed an increase in *in vitro* microbial mass yield (106%–681%), *in vitro* dry matter disappearance (IVDMD) (40.9%–240%), and total volatile fatty acids (VFA, 5.85%–11.2%). In another *in vitro* experiment, Ngaowkakhiaw et al. [9] stated that SMS ensiled with whole corn at 1:1 decreased methane (CH_4_) emission and reduced DM disappearance (*d*DM).

*In vitro* batch culture technology is widely used in animal nutrition and microbiology research, particularly to investigate feed digestion in the rumen of livestock under controlled conditions [13],[14]. Recent improvements in batch culture protocols have enhanced the accuracy and consistency of *in vitro* studies by refining key elements such as temperature, pH, and gas composition, allowing for a more precise simulation of the rumen environment and thus increasing the reliability of findings compared to *in vivo* studies [15],[16]. This technology facilitates rapid assessments of new feeds, ruminal CH_4_ reduction strategies, the effects of new feed supplements, and the microbial interactions within the rumen that affect fermentation efficiency and nutrient absorption [17].

Limited information is available on the optimal proportions and the most suitable storage length of spent mushroom substrate when included as an additive and ensiled with corn silage (CS). We hypothesize that CS containing a combination of spent mushroom substrates will produce silages with increased nutritive value for ruminants. Therefore, the objective of this study is to determine the effect of varying proportions of spent mushroom (*P. ostreatus*) substrate (at 10%, 20%, 30%, 40%, and 50%) and ensiling duration (at 0, 21, 42, and 64 d) on *in vitro* dry matter digestibility, total short-chain fatty acids, and greenhouse gas (GHG) emissions of CS.

## 2. Materials and methods

### 2.1. Silage preparation

The experiment was conducted at the Department of Animal Sciences at North Carolina Agricultural and Technical State University. Corn was manually harvested to a stubble of 15 cm from the University farm when the kernel was at approximately 1/3 the milk line stage and chopped to approximately 2 cm with a forage chopper. The spent mushroom substrates were generated from corn stover inoculated with *P. ostreatus* grain spawn (5% wet weight) as described in Olagunju et al. [6]. The corn was ensiled in a plastic bucket with a 5 L capacity (cylindrical shape with 35 cm height × 16 cm diameter), and each storage time group was prepared with four replications. The ensiling was done within 6 h after harvesting. The buckets were stored in a room at an ambient temperature of 20 ± 2 °C for 0, 21, 42, and 63 d. Three-week intervals were selected during ensiling to determine the optimal period to maximize enzymatic activity and fiber degradation. Spent mushroom substrate was thoroughly mixed with the CS at different ratios before ensiling. Subsamples were dried at 60 °C for 48 h in a forced-air oven and milled to pass through 1 mm sieve size for DM determination.

### 2.2. Experimental design

The study was a 6 × 4 factorial design with 6 inclusion levels and 4 ensiling durations. The varying proportions (CS:SMS) were 100:0 (CS100:SMS0), which served as the control group, 90:10 (CS90:SMS10), 80:20 (CS80:SMS20), 70:30 (CS70:SMS30), 60:40 (CS60:SMS40), and 50:50 (CS50:SMS50). The ensiling durations were 0, 21, 42, and 63 d. Inclusion of SMS from 0% (control) to 50% is a practical approach to evaluate its potential to reduce feed cost. A total of four replicates were prepared for each treatment consisting of 24 treatments (6 inclusion levels and 4 lengths of storage) and 96 mini bucket silos (4 replications per treatment).

### 2.3. Sample preparation

Silos were weighed after filling and at opening to determine DM recovery. After opening, the silages were mixed and samples were collected for analysis of chemical composition, *in vitro* batch culture, GHG emissions, and volatile fatty acid determination. Milled silage samples (0.5 ± 0.02 g) were weighed with an analytical scale (model VWR-224AC; VWR International, Radnor, PA, USA) directly into 100 mL serum bottles (Cat# 223747; Wheaton Science Products, Millville, NJ, USA). Four replicates (bottles) were prepared for each treatment and incubated for 24 h. For determination of DM digestibility, 0.5 g of the samples was weighed into Ankom bags (F57; Ankom Technology Corp, Macedon, NY, USA) and sealed using a heat impulse sealer (Model# AIE-200HR, California, USA) before the bags were inserted into pre-labeled 100 mL serum bottles according to treatments, with four replicates per treatment.

### 2.4. In vitro batch culture

The *in vitro* incubation procedure was based on the methods described by Anele et al. [18] with some modifications. First, Ankom bags (Ankom Technology Corp., Macedon, NY, USA) were labeled, washed with acetone, and weighed. The bags were filled with approximately 0.5 ± 0.052 g of feed samples and sealed with an impulse heat sealer. Once sealed, bags were placed into the respective 100 mL serum bottles. Artificial saliva was prepared according to McDougall's buffer recipe containing (per L): 9.83 g NaHCO_3_, 3.69 g Na_2_HPO_4_, 0.60 g KCl, 0.47 g NaCl, 0.30 g (NH_4_)_2_SO_4_, 0.061 g MgCl_2_·6H_2_O, and 0.0293 g CaCl_2_·2H_2_O. The artificial saliva was maintained in a water bath at 39 °C. Ruminal fluid was collected from two ruminally cannulated Holstein Friesian dairy cows after morning feeding. Cows were maintained on pasture and supplemented with grass hay and a mineral premix. The cows were from the Dairy Research Farm of North Carolina Agricultural and Technical State University. The artificial saliva and ruminal fluid were mixed at 3:1 (v/v) and the pH was measured using a bench top pH meter (model B10P, VWR International, Randor, PA, USA). The batch culture media was dispensed into the 100 mL glass serum bottles. Each serum bottle received 45 mL of artificial saliva and 15 mL of rumen fluid anaerobically by flushing with carbon dioxide (CO_2_). The bottles were capped with a 14 mm rubber stopper and crimped with an aluminum seal cap. The bottles were incubated on an orbital shaker at 39 °C at a speed of 125 rpm for 6 and 24 h. This process was repeated on a different week for a total of two runs. At the pre-determined time points, headspace gas production was measured at 6 and 24 h post incubation by inserting a 22 mm gauge needle attached to a manometer (VWR International, Randor, PA, USA).

### 2.5. Estimation of greenhouse gases

Methane, ammonia (NH_3_), CO_2_, and hydrogen sulfide (H_2_S) concentrations were estimated using a portable gas analyzer (Biogas 5000, Landtec, Dexter, MI, USA). The analyzer was calibrated according to the manufacturer's instructions. An aliquot of gas from the samples was introduced into the analyzer with a 22 G × 1 ½ (0.7 mm × 40 mm) gauge needle attached to the end of the inlet Tygon tube. The unit was purged between each sampling to eliminate any residual gas from the previous sampling.

### 2.6. Nutrient disappearance

After gas pressure and analysis readings, the Ankom bags were removed from the bottles, thoroughly rinsed under cold water until the water was clear, and dried in a 55 °C oven for 48 h. Thereafter, IVDMD was estimated as described by Anele et al. [19]. Dry matter (*d*DM), NDF (*d*NDF), ADF (*d*ADF), and ADL (*d*ADL) disappearance were calculated by subtracting the residual weight from the initial weight of the substrate.

### 2.7. Microbial mass estimation

Microbial mass determination was based on Blümmel and Lebzien [20] with a slight modification. Feed substrates were weighed directly into the serum bottles, and fermentation was terminated after 24 h. The contents of the bottles were poured into pre-weighed centrifuge tubes (Thermo Fisher Scientific, Rochester, NY, USA). The samples were centrifuged at 20,000 × g at 4 °C for 15 min. Blanks were centrifuged as a correction factor for the buffered ruminal inoculum residues. After removing the tubes from the centrifuge, the supernatant was poured off, and the pellets were placed in a freezer for 24 h. The frozen pellets were placed in a freeze-dryer (L-200, BUCHI Lyovapor, New Castle, DE, USA) for approximately 72 h. After freeze-drying, the lyophilized samples were weighed to determine the weight of the pellets and microbial mass was calculated as described by Blümmel and Lebzien [20]. The partitioning factor (PF_24_) at 24 h of incubation (mg *d*DM: mL gas) was calculated [21].

### 2.8. Volatile fatty acid analysis

Volatile fatty acid concentration was measured based on the protocol of Ruiz-Moreno et al. [22]. The samples were analyzed using a gas chromatography (Agilent 7890B GC system/5977B GC-MSD/7693 autosampler, Agilent Technologies, Santa Clara, CA, USA) with a capillary column (Zebron ZB-FFP, Phenomenex Inc., Torrance, CA, USA). An internal standard mixture of metaphosphoric acid and crotonic acid (trans-2-butenoic acid) was employed, while acetate (C_2_), propionate (C_3_), butyrate (C_4_), isobutyrate (iso-C_4_), valerate (C_5_), and isovalerate (iso-C_5_) served as quantitative external standards [22].

### 2.9. Chemical analysis

The dietary samples were analyzed for DM (#930.15), N (#954.01), and ether extract (EE; #920.39) according to AOAC [23]. Nitrogen was determined using an organic elemental analyzer (2400 CHNS, PerkinElmer, Waltham, MA, USA). Ether extract was determined using the Ankom XT15 (Ankom, Macedon, NY, USA) extractor. Neutral-detergent fiber (NDF) and acid-detergent fiber (ADF) were analyzed using Ankom 200 Fiber Analyzer (Ankom, Macedon, NY, USA). The NDF content was determined as described by Van Soest et al. [24] using heat stable α-amylase with sodium sulfite. Acid-detergent fiber was determined according to AOAC [23] (method 973.18). Acid-detergent lignin (ADL) was determined by soaking in concentrated sulfuric acid using ANKOM Technologies analytical methods. The concentrations of non-structural carbohydrates (NSC = 1000 − NDF − CP − EE − ash), cellulose (ADF-ADL), hemicellulose (NDF-ADF), and organic matter (OM = 1000 − ash) in feed ingredients and feces were calculated.

### 2.10. Statistical analysis

Data generated were analyzed using the PROC MIXED procedure of SAS 9.4 (SAS Inst., Inc., Cary, NC, USA) in a 6 × 4 factorial arrangement. The model used was: Y_ijk_ = μ + P_i_ + S_j_ + (PS)_ij_ + E_ijk_, where Y_ijk_ is each observation for a given variable, μ is the overall mean, P_i_ is the proportion effect, S_j_ is the effect of ensiling duration, (PS)_ij_ is the interaction between proportion and ensiling duration, and E_ijk_ is the residual error. The probability of difference option of the least squares means the statement was used for multiple comparisons of means, and polynomial (linear and quadratic) contrasts (adjusted for the equal spacing of ensiling duration) were used to examine duration responses to increasing the ensiling duration. Significance was declared at a level of P < 0.05.

## 3. Results

### 3.1. Chemical composition

Significant interactions (P < 0.01) between duration and diet were observed for the concentrations of the different nutrients (Table 1). Diets significantly (P < 0.001) affected the concentrations of all the nutrients. Increases in the proportion of SMS in the diets generally increased (P < 0.001) the concentrations of DM, NDF (except CS90:SMS10), ADF (except CS90:SMS10), ADL, and hemicellulose; however, a decrease in the concentrations of CP, EE, NSC (except CS80:SMS20 and CS90:SMS10), and cellulose was also noted with increasing SMS proportion in the diets.

Ensiling duration affected (P < 0.01) the concentrations of DM (linear and quadratic), OM (quadratic), CP (quadratic), NSC (linear), NDF (linear and quadratic), ADF (quadratic), hemicellulose (linear and quadratic), and cellulose (linear and quadratic). With increasing ensiling duration, the concentrations of DM, NDF, ADL (linear effect), and hemicellulose (linear and quadratic effects, P < 0.001) were decreased. Also, OM concentration was decreased (quadratic effect, P < 0.001) in all the diets except in CS100:SMS0 and CS80:SMS20, and CP at 42 and 63 days in CS100:SMS0, 21 and 42 days in the CS90:SMS10 diet, and 63 days in the CS70:SMS30, CS60:SMS40, and CS50:SMS50 diets. Increasing the ensiling duration increased (P < 0.05) NSC, ADF (except in CS100:SMS0 and CS90:SMS10 diets), and cellulose (except in CS100:SMS0).

### 3.2. Gas and greenhouse gases

Significant interactions between duration and diet were observed (P = 0.008) for GP and CO_2_ production (Table 2). Diet linearly affected (P < 0.01) the production of gas and individual GHG. Increasing the proportion of SMS decreased (P < 0.001) GP, CH_4_, CO_2_ (except in CS80:SMS20), NH_3_ (except in CS90:SMS10), and H_2_S (except in CS80:SMS20 and CS90:SMS10 diets).

Ensiling duration linearly affected (P < 0.01) total gas production and individual GHG. Increasing the ensiling duration linearly and quadratically increased (P < 0.01) GP, CH_4_, and CO_2_, and quadratically decreased NH_3_ production (P = 0.003) from all the diets except CS80:SMS20 at 42 days and CS80:SMS20 at 42 and 63 days. Moreover, increasing the ensiling duration decreased H_2_S production in CS60:SMS40. The CS100:SMS0 diet had the highest GP, CH_4_, CO_2_, NH_3_, and H_2_S production at 63 days compared to the other diets. The lowest CH_4_ and CO_2_ concentrations were observed in CS90:SMS10 at 0 days, while CS50:SMS50 and CS80:SMS20 diets at 21 days had the lowest NH_3_ and H_2_S concentrations.

**Table 1.** Chemical composition (%, DM basis) of diets with different spent mushroom substrate (SMS) and corn silage (CS) proportions during different ensiling durations (days).

Diet^1^	Days	DM	OM	CP	EE	NSC	NDF	ADF	ADL	HC	CL
CS100:SMS0	0	97.9	93.7	12.2	2.46	36.7	44.8	26.1	3.43	18.6	22.7
	21	97.0	93.7	15.8	1.61	36.7	41.2	24.6	7.03	16.6	17.6
	42	97.8	94.6	9.8	2.60	42.9	42.0	26.3	2.66	15.7	23.6
	63	97.2	93.8	6.5	4.44	47.5	39.9	23.9	3.30	15.9	20.9
CS90:SMS10	0	99.1	93.1	7.0	1.33	29.3	56.8	31.8	10.46	25.0	21.3
	21	97.0	90.3	6.3	1.74	34.3	49.8	34.5	9.76	15.3	24.7
	42	98.1	91.8	5.9	1.59	33.9	52.0	34.7	7.37	17.3	27.3
	63	98.1	92.2	9.1	1.08	29.5	53.6	33.8	8.00	19.8	25.8
CS80:SMS20	0	99.0	93.1	7.7	1.52	31.1	54.3	32.0	10.33	22.3	21.6
	21	97.7	92.2	6.8	1.89	39.8	45.6	29.0	6.61	16.6	22.3
	42	98.3	93.1	9.6	2.00	37.6	45.9	30.0	5.68	15.9	24.4
	63	98.2	93.2	9.0	0.94	35.2	48.9	30.3	5.83	18.7	24.4
CS70:SMS30	0	98.8	93.6	8.1	1.83	34.9	50.6	27.9	9.32	22.6	18.6
	21	97.9	92.9	8.1	1.19	38.3	46.5	28.5	5.97	18.0	22.6
	42	97.9	93.3	11.8	2.28	38.1	43.4	28.0	5.07	15.4	22.9
	63	97.8	93.1	7.3	2.45	38.3	47.5	29.5	4.85	18.0	24.7
CS60:SMS40	0	98.3	93.7	7.6	1.87	38.3	47.9	27.1	6.20	20.7	20.9
	21	97.4	92.6	8.0	1.58	40.6	44.0	28.2	5.58	15.8	22.6
	42	97.5	93.3	16.0	2.17	31.6	45.6	29.1	4.02	16.5	25.1
	63	97.3	93.5	6.8	2.19	42.4	44.2	27.0	4.35	17.3	22.6
CS50:SMS50	0	98.3	94.7	8.4	2.28	42.7	43.6	24.0	4.09	19.6	19.9
	21	96.8	92.9	13.5	1.21	37.7	41.7	26.8	4.56	14.9	22.2
	42	96.1	92.5	10.6	2.48	39.3	42.5	27.2	3.84	15.3	23.4
	63	97.2	93.6	5.8	1.17	45.4	42.4	25.7	3.86	16.7	21.9
Pooled SEM		0.220	0.218	0.666	0.394	0.879	0.470	0.620	0.780	0.555	0.987
Pooled *P* value										
Diet		<0.001	<0.001	<0.001	<0.001	<0.001	<0.001	<0.001	<0.001	<0.001	<0.001
Ensiling duration										
Linear		<0.001	0.125	0.051	0.115	<0.001	<0.001	0.270	<0.001	<0.001	<0.001
Quadratic		<0.001	<0.001	<0.001	0.530	0.908	<0.001	0.013	0.132	<0.001	0.008
Duration × Diet		0.008	<0.001	<0.001	0.001	<0.001	<0.001	<0.001	0.004	<0.001	0.003

ADF is acid-detergent fiber, ADL is acid-detergent lignin, CL is cellulose, CP is crude protein, DM is dry matter, EE is ether extract, HC is hemicellulose, NDF is neutral-detergent fiber, NSC is non-structural carbohydrates, and OM is organic matter. ^1^Spent mushroom substrate (SMS) was mixed with corn silage (CS) at 100:0 (control group; CS100:SMS0), 90:10 (CS90:SMS10), 80:20 (CS80:SMS20), 70:30 (CS70:SMS30), 60:40 (CS60:SMS40), and 50:50 (CS50:SMS50).

**Table 2.** Gas and greenhouse gases produced from diets with different spent mushroom substrate (SMS) and corn silage (CS) proportions during different ensiling durations (days).

Diet^1^	Days	GP	CH_4_	CO_2_	NH_3_	H_2_S
CS100:SMS0	0	147.9	6.68	31.4	96.1	364.0
	21	159.2	7.52	40.9	94.5	382.5
	42	176.4	7.96	44.4	92.0	442.5
	63	184.5	8.64	51.1	118.9	512.2
CS90:SMS10	0	106.1	4.49	25.4	72.0	283.8
	21	119.0	5.56	30.9	52.2	212.0
	42	119.9	4.61	28.6	74.7	327.6
	63	111.5	4.49	29.0	63.9	290.1
CS80:SMS20	0	105.9	4.33	23.9	74.8	272.0
	21	128.6	5.76	33.1	49.0	148.6
	42	137.4	5.16	31.0	79.3	327.7
	63	129.0	5.65	35.6	75.6	340.3
CS70:SMS30	0	124.2	5.43	29.7	87.4	323.9
	21	129.8	6.00	33.4	52.3	205.4
	42	153.2	6.24	37.4	85.9	365.1
	63	139.7	5.54	33.3	75.4	318.4
CS60:SMS40	0	133.0	6.04	31.9	92.8	385.1
	21	143.5	6.77	37.5	58.2	186.1
	42	163.6	7.18	40.2	83.9	339.2
	63	160.5	6.84	40.1	91.2	382.8
CS50:SMS50	0	141.5	4.90	21.2	103.6	391.9
	21	159.9	8.01	42.7	82.7	318.0
	42	169.4	6.98	43.0	99.4	425.2
	63	171.6	8.54	50.0	104.7	474.2
Pooled SEM		3.732	0.461	2.567	10.013	50.709
Pooled P value					
Diet		<0.001	<0.001	<0.001	<0.001	<0.001
Ensiling durations					
Linear		<0.001	<0.001	<0.001	0.022	0.004
Quadratic		<0.001	0.008	0.002	0.003	0.010
Duration × Diet		0.008	0.069	0.008	0.864	0.910

CH_4_ is methane at 24 h of incubation (mg/g DM), CO_2_ is carbon dioxide at 24 h of incubation (mg/g DM), GP is gas production (mL/g DM), H_2_S is hydrogen sulfide at 24 h of incubation (mmol/g DM), NH_3_ is ammonia (mmol/g DM). ^1^Spent mushroom substrate (SMS) was mixed with corn silage (CS) at 100:0 (control group; CS100:SMS0), 90:10 (CS90:SMS10), 80:20 (CS80:SMS20), 70:30 (CS70:SMS30), 60:40 (CS60:SMS40), and 50:50 (CS50:SMS50).

### 3.3. Nutrient disappearance, partitioning factor, and microbial mass

No significant interactions between duration and diet were observed for nutrient degradability, PF24, or MM (Table 3). However, diet affected nutrient degradability and PF24. Increasing the proportion of SMS in the diets increased dDM, dNDF (except in CS80:SMS20 and CS90:SMS10), dADF, dADL, undegraded residuals, and PF24. Similarly, MM (except in CS80:SMS20 and CS90:SCS10 diets) decreased with increasing SMS inclusion in the diets.

**Table 3.** Nutrient degradability (%), undegraded residual (g), dry matter disappearance, partitioning factor, and microbial mass (g) of diets with different spent mushroom substrate (SMS) and corn silage (CS) proportions during different ensiling durations (days).

Diet^1^	Days	*d*DM	*d*NDF	*d*ADF	*d*ADL	Undegraded	PF_24_	MM
CS100:SMS0	0	42.0	77.2	59.3	18.9	0.21	1.85	0.052
	21	44.0	73.2	61.3	19.4	0.22	1.85	0.142
	42	41.4	76.5	59.5	14.6	0.23	1.51	0.005
	63	44.0	74.1	58.8	19.2	0.20	1.61	0.016
CS90:SMS10	0	33.7	79.6	63.1	30.1	0.26	2.25	0.032
	21	33.4	76.6	65.0	32.1	0.26	2.07	0.125
	42	34.6	77.6	63.7	24.5	0.27	2.06	0.019
	63	35.4	78.1	63.9	23.5	0.24	2.24	0.030
CS80:SCS20	0	34.7	79.3	62.7	35.9	0.24	2.33	0.052
	21	37.6	73.0	64.8	27.9	0.25	2.09	0.164
	42	40.9	76.7	61.8	18.6	0.25	1.94	0.012
	63	39.2	77.6	61.3	24.6	0.22	2.13	0.039
CS70:SMS30	0	38.0	78.5	61.5	25.8	0.24	2.16	0.044
	21	38.7	75.0	62.4	30.1	0.24	2.02	0.115
	42	41.5	75.9	59.9	23.9	0.24	1.78	0.009
	63	39.2	77.0	59.7	19.1	0.21	1.93	0.036
CS60:SMS40	0	39.2	77.0	61.4	23.1	0.22	2.12	0.058
	21	40.1	72.6	62.6	25.7	0.23	1.85	0.091
	42	40.8	78.8	60.7	22.4	0.24	1.62	0.005
	63	43.6	77.9	59.7	17.1	0.21	1.73	0.021
CS50:SMS50	0	40.9	72.6	60.1	23.8	0.21	2.07	0.059
	21	41.7	74.1	61.5	23.4	0.22	1.78	0.131
	42	43.1	77.9	58.7	21.9	0.23	1.62	0.005
	63	43.0	75.2	57.9	16.5	0.20	1.65	0.020
Pooled SEM		1.133	1.225	1.104	2.621	0.005	0.063	0.0222
Pooled P value							
Diet		<0.001	0.010	<0.001	<0.001	<0.001	<0.001	0.001
Ensiling durations							
Linear		<0.001	0.654	0.006	<0.001	<0.001	<0.001	<0.001
Quadratic		0.376	0.008	0.022	0.585	<0.001	<0.001	<0.001
Duration × Diet		0.248	0.053	0.998	0.127	0.756	0.053	0.273

*d*DM is dry matter degradability, *d*NDF is neutral-detergent fiber degradability, *d*ADF is acid-detergent fiber degradability, *d*ADL is acid-detergent lignin degradability, PF_24_ is partitioning factor (mg *d*DM: mL gas), and MM is microbial mass (g/kg DM). ^1^Spent mushroom substrate (SMS) was mixed with corn silage (CS) at 100:0 (control group; CS100:SMS0), 90:10 (CS90:SMS10), 80:20 (CS80:SMS20), 70:30 (CS70:SMS30), 60:40 (CS60:SMS40), and 50:50 (CS50:SMS50).

Ensiling duration linearly affected *d*DM and *d*ADL, quadratically affected *d*NDF and *d*ADF, and linearly and quadratically affected undegraded residuals, PF_24_, and MM. With increasing ensiling duration, there was an increase in *d*DM (except in CS100:SMS0 at 42 days and CS90:SMS10 at 21 days), PF_24_, and undegraded residuals (at all days except 63 days). However, increasing the ensiling duration decreased *d*NDF (except in CS60:SMS40 and CS50:SMS50 diets), *d*ADF (except in CS90:SMS10 diet), *d*ADL (except in CS90:SMS10 at 21 days, CS70:SMS30 at 21 days, and CS60:SMS40 at 21 days) and MM at 42 and 63 days. Both CS60:SMS40 and CS50:SMS50 diets at 0 days had the highest *d*NDF and *d*ADF, while the lowest *d*NDF was observed in CS90:SMS10 at 0 days and CS80:SMS20 at 21 days, while CS90:SMS10 at 63 days had the lowest *d*ADF.

### 3.4. Volatile fatty acids

No significant interactions between duration and diet were observed for total and individual VFA (Table 4). Except for total VFA (P = 0.013) and butyrate proportion (P = 0.002), diet did not affect the concentrations of individual VFA. As the proportion of SMS increased in the diet, the concentration of total VFA (P = 0.013) (except in CS90:SMS10) and C_4_ proportion (P = 0.002) (except in CS90:SMS10 and CS80:SMS20 diets) decreased. The CS90:SMS10 diet had the highest total VFA concentration and butyrate proportion.

Ensiling duration linearly and quadratically affected (P < 0.05) total VFA, C_4_, and iso-C_4_, and quadratically affected C_5_ and iso-C_5_, while linearly affecting C_2_ and C_3_ proportions. Ensiling for 42 and 63 days decreased total VFA in CS100:SMS0, CS90:SMS10, and CS70:SMS30 diets, and increased total VFA concentration was observed in CS60:SMS40 diet at 21 and 42 days. Increasing the ensiling duration linearly increased C_2_ (P = 0.043) in all the diets and C_3_ (P = 0.004) except at 21 days, and decreased C_4_ and iso-C_4_ proportions (P < 0.05). The highest C_5_ and iso-C_5_ proportions were observed (quadratic effects, P < 0.01) in CS90:SMS10 at 63 days and CS50:SMS50 at 0 days.

### 3.5. Correlation between chemical composition and measured parameters

Positive and negative correlations (P < 0.05 and P < 0.01) were observed between nutrient concentrations and measured parameters (Table 5).

**Table 4.** Total (mmol/L) and individual volatile fatty acids (%) produced from diets with different spent mushroom substrate (SMS) and corn silage (CS) proportions during different ensiling durations (days).

Diet^1^	Days	Total	C_2_	C_3_	C_2_:C_3_	C_4_	C_5_	Iso-C_4_	Iso-C_5_
CS100:SMS0	0	73.0	59.8	20.5	3.06	17.7	0.37	1.49	0.20
	21	94.6	62.5	21.3	3.55	14.6	0.29	1.17	0.15
	42	69.8	62.1	24.8	2.51	11.9	0.35	0.72	0.19
	63	64.7	61.7	25.1	2.46	11.9	0.34	0.73	0.19
CS90:SMS10	0	58.8	63.9	20.3	3.22	14.0	0.37	1.25	0.21
	21	73.8	68.2	18.3	4.70	12.0	0.29	0.93	0.15
	42	51.1	65.7	22.0	2.99	11.1	0.37	0.66	0.19
	63	47.6	64.0	22.5	2.85	12.1	0.41	0.80	0.22
CS80:SMS20	0	60.3	63.0	20.1	3.25	15.1	0.38	1.31	0.22
	21	76.0	66.5	19.3	4.40	12.7	0.28	1.09	0.15
	42	60.6	63.8	23.4	2.73	11.5	0.37	0.69	0.19
	63	66.2	67.4	21.0	3.42	10.4	0.33	0.65	0.18
CS70:SMS30	0	65.6	60.0	20.8	3.00	17.2	0.39	1.43	0.22
	21	78.7	64.5	19.7	3.89	14.3	0.30	1.08	0.15
	42	55.5	62.4	24.2	2.58	12.1	0.38	0.73	0.20
	63	60.3	63.4	23.9	2.65	11.5	0.35	0.69	0.19
CS60:SMS40	0	71.3	58.4	21.3	2.80	18.1	0.39	1.54	0.22
	21	74.6	63.1	21.2	3.08	14.3	0.29	1.00	0.14
	42	71.7	63.9	23.1	2.83	11.7	0.36	0.75	0.20
	63	62.8	62.6	24.2	2.59	11.9	0.36	0.74	0.20
CS50:SMS50	0	81.3	56.2	21.9	2.79	19.5	0.41	1.78	0.23
	21	84.4	59.2	23.6	2.55	15.5	0.33	1.28	0.17
	42	84.7	65.3	22.5	3.30	11.0	0.34	0.69	0.18
	63	60.1	61.9	24.6	2.52	12.3	0.36	0.73	0.19
Pooled SEM		8.371	2.976	1.896	0.570	0.831	0.035	0.170	0.020
Pooled P value								
Diet		0.013	0.057	0.237	0.464	0.002	0.915	0.663	0.930
Ensiling durations								
Linear		0.010	0.043	0.004	0.130	<0.001	0.902	<0.001	0.509
Quadratic		0.012	0.056	0.730	0.142	<0.001	0.002	0.011	<0.001
Duration × Diet		0.870	0.959	0.967	0.949	0.252	0.991	0.994	0.997

C_2_ is acetate, C_3_ is propionate, C_4_ is butyrate, and C_5_ is valerate (mmol/g DM). ^1^Spent mushroom substrate (SMS) was mixed with corn silage (CS) at 100:0 (control group; CS100:SMS0), 90:10 (CS90:SMS10), 80:20 (CS80:SMS20), 70:30 (CS70:SMS30), 60:40 (CS60:SMS40), and 50:50 (CS50:SMS50).

**Table 5.** Pearson correlation between *in vitro* fermentation products and nutrient contents mean from diets with different spent mushroom substrate (SMS) and corn silage (CS) proportions during different ensiling durations (days).

	GP	CH_4_	CO_2_	NH_3_	H_2_S	VFA	C_2_	C_3_	C_2_:C_3_	C4	Iso-C_4_	C_5_	Iso-C_5_	*d*DM	*d*NDF	*d*ADF	*d*ADL	MM	PF_24_
DM	−0.57**	−0.51**	−0.51**	−0.13	−0.14	−0.16	0.01	−0.20*	0.06	0.19*	0.16	0.20*	0.19*	−0.38**	0.25**	0.18*	0.17*	−0.25**	0.49
OM	0.35**	0.19*	0.09	0.36**	0.31**	0.12	−0.23**	0.13	−0.17	0.26**	0.14	0.16	0.17	0.39**	−0.03	−0.34**	−0.34**	−0.27**	−0.23**
CP	0.29**	0.23**	0.14	0.14	0.10	0.24**	−0.07	0.05	−0.04	0.06	−0.05	0.04	−0.07	0.30**	−0.01	−0.11	−0.14	0.22*	−0.27**
EE	0.34**	0.22**	0.21*	0.22**	0.22**	−0.03	−0.10	0.19*	−0.12	−0.02	0.05	−0.05	0.04	0.18*	−0.09	−0.21*	−0.14	−0.09	−0.31**
NSC	0.64**	0.48**	0.45**	0.25**	0.25**	0.13	−0.15	0.24**	−0.12	0.00	−0.06	−0.09	−0.08	0.52**	−0.36**	−0.37**	−0.35**	−0.04	−0.51**
NDF	−0.84**	−0.66**	−0.57**	−0.30**	−0.27**	−0.28**	0.15	−0.27**	0.13	0.02	0.13	0.10	0.18	−0.70**	0.40**	0.41**	0.42**	−0.15	0.70**
ADF	−0.64**	−0.51**	−0.38**	−0.37**	−0.29**	−0.25**	0.29**	−0.19*	0.15	−0.30**	0.00	−0.15	−0.01	−0.62**	0.31**	0.44**	0.35**	−0.04	0.43**
ADL	−0.64**	−0.44**	−0.39**	−0.24**	−0.26**	−0.12	0.05	−0.20*	0.10	0.11	0.05	0.17	0.06	−0.44**	0.23**	0.40**	0.40**	0.09	0.58**
Hemi	−0.58**	−0.44**	−0.46**	−0.04	−0.09	−0.15	−0.09	−0.20*	0.03	0.36**	0.21	0.33**	0.28**	−0.39**	0.27**	0.15	0.25**	−0.19*	0.60**
Cell	−0.07	−0.14	−0.04	−0.15	−0.05	−0.15	0.25**	0.00	0.05	−0.43**	−0.06	−0.33**	−0.07	−0.24**	0.13	0.10	−0.01	−0.15	−0.09
GP	1.00	0.78**	0.69**	0.45**	0.38**	0.10	−0.05	0.26**	−0.14	−0.18*	−0.13	−0.28**	−0.14	0.69**	−0.26**	−0.40**	−0.55**	−0.09	−0.85**
CH_4_		1.00	0.94**	0.45**	0.41**	0.05	0.05	0.08	0.00	−0.14	−0.21	−0.25**	−0.21*	0.52**	−0.27**	−0.20*	−0.44**	0.14	−0.66**
CO_2_			1.00	0.40**	0.51**	0.11	0.02	0.25**	−0.09	−0.29**	−0.11	−0.25**	−0.14	0.43**	−0.18*	−0.24**	−0.38**	−0.01	−0.60**
NH_3_				1.00	0.70**	−0.12	0.08	−0.09	0.04	0.01	−0.17	−0.21*	−0.10	0.23**	0.01	−0.31**	−0.27**	−0.20*	−0.38**
H_2_S					1.00	0.14	−0.23	0.33**	−0.24**	0.00	0.20	0.12	0.21*	0.34**	0.12	−0.36**	−0.29**	−0.33**	−0.32**
VFA						1.00	0.03	−0.08	0.30**	−0.02	−0.08	0.35**	−0.10	0.16	−0.18*	−0.09	0.04	0.28**	−0.05
C_2_							1.00	−0.82**	0.87**	−0.76**	−0.85	−0.77**	−0.80**	−0.31**	0.10	0.19	0.06	0.23	−0.10
C_3_									−0.90**	0.25**	0.75	0.40**	0.66**	0.41**	−0.02	−0.29**	−0.17	−0.42**	−0.17
C_2_:C_3_									1.00	−0.46**	−0.76	−0.44**	−0.69**	−0.30**	−0.05	0.23*	0.11	0.41**	0.05
C4										1.00	0.55	0.80**	0.58**	0.07	−0.15	−0.02	0.07	0.10	0.32**
Iso-C_4_											1.00	0.69**	0.97**	0.23**	0.00	−0.03	−0.11	−0.48**	0.27**
C_5_												1.00	0.68**	0.08	−0.08	0.01	0.16	0.02	0.41**
Iso-C_5_													1.00	0.19*	−0.02	−0.02	−0.13	−0.48**	0.30**
*d*DM														1.00	−0.19*	−0.26**	−0.54**	−0.14	−0.48**
*d*NDF															1.00	−0.22**	0.26**	−0.28**	0.03
*d*ADF																1.00	0.02	0.30**	0.43**
*d*ADL																	1.00	0.22*	0.41**
MM																		1.00	0.08
PF_24_																			1.00

*P < 0.05, **P < 0.01. ADF is acid-detergent fiber, ADL is acid-detergent lignin, CL is cellulose, CP is crude protein, DM is dry matter, EE is ether extract, HC is hemicellulose, NDF is neutral-detergent fiber, NSC is non-structural carbohydrates, OM is organic matter, CH_4_ is methane at 24 h of incubation (mg/g DM), CO_2_ is carbon dioxide at 24 h of incubation (mg/g DM), GP is gas production (mL/g DM), H_2_S is hydrogen sulfide at 24 h of incubation (mmol/g DM), NH_3_ is ammonia (mmol/g DM), *d*DM is dry matter degradability, *d*NDF is neutral-detergent fiber degradability, *d*ADF is acid-detergent fiber degradability, *d*ADL is acid-detergent lignin degradability, PF_24_ is partitioning factor (mg degradable DM: mL gas), MM is microbial mass (g/kg DM), C_2_ is acetate, C_3_ is propionate, C_4_ is butyrate, and C_5_ is valerate (mmol/g DM).

## 4. Discussion

### 4.1. Chemical composition

The significant interaction between ensiling duration and diet indicates that nutrient concentrations are diet and duration dependent. Nutrient concentration differed between diets since both CS and SMS had different chemical compositions. Increases in the proportion of SMS in the diets increased the concentrations of DM, NDF, ADF, ADL, and hemicellulose in almost all the diets with a few exceptions. Such results were expected since we replaced CS with SMS, which was obtained after the harvest of mushroom fruits. Fungi get their requirements from breaking lignocellulolytic constituents through the production of exogenous enzymes such as lignin peroxidase, manganese peroxidase, H_2_O_2_ producer enzymes, arylachol oxidase, and laccase [25]. Therefore, high proportions of such nutrients were utilized by the mushroom for fruit production and would explain why the concentrations of fiber fractions were increased in the SMS compared to CS [8],[26],[27]. Akinfemi and Ogunwole [28], Khattab et al. [26], and Olagunju et al. [6] reported that SMS had higher concentrations of fiber than the original substrate before mushroom cultivation. Adamovic et al. [29] reported that values for NDF, ADF, hemicellulose, cellulose, and lignin linearly increased when spent *P. ostreatus* mushroom substrate was included in silage (from 10% to 30%). However, Kwak et al. [27] demonstrated that cotton waste–based SMS ensiled with molasses, lactic acid bacteria, or yeast for 10 days exhibited minimal changes in chemical composition.

The use of this kind of silage in smaller quantities (up to 10% of DM in diets for cows and fattening bulls) could be reasonable in diets with low ADF and NDF content, as well as for cattle with lower genetic potential, or under extensive management system [29].

Combining SMS with other ingredients significantly improves its nutrient profile and overall nutritive value before it is fed to animals [30]–[32]. Increasing the proportion of SMS in the diet decreased the concentrations of CP, EE, NSC, and cellulose in almost all the diets. *P. ostreatus* mushrooms are unable to get their carbon and energy requirements from only lignin and therefore require substrates such as cellulose or other carbon sources for their growth and de-lignification [25]. In an *in vitro* experiment, Ngaowkakhiaw et al. [9] reported that mixing SMS with CS at 1:1 DM basis decreased the concentration of hemicellulose, NSC, and CP. However, Olagunju et al. [6] observed that corn stover treated with P. *ostreatus* had higher CP concentration compared to untreated corn stover. They reported that their result could be due to an efficient conversion of nitrogen to microbial protein by *P. ostreatus*.

Increasing ensiling duration decreased the concentrations of DM, OM, NDF, ADL, and hemicellulose, which may be due to their utilization by the microbes during the ensiling process. The effect of ensiling duration on CP varied among the diets, increasing or decreasing on different days. The observed variation did not follow any regular pattern so one can only speculate on the actual cause of the variation. Diets containing SMS at 0%, 20%, 30%, 40%, and 50% had higher CP concentrations compared to the 10% SMS diet.

Increasing the ensiling days increased the concentrations of NSC, ADF, and cellulose in most diets. Darwish et al. [33] reported that a single *P. ostreatus* treatment of maize stalks gave lower protein content when compared to simultaneously double-treated maize stalk with *P. ostreatus* and *Saccharomyces cerevisiae*, while cellulose, hemicellulose, and lignin had a gradual decrease with increasing incubation time. The nutritive value of rice straw treated with *P. ostreatus*, *P. pulmonarius*, and *P. tuber-regium* had higher CP but lower crude fiber cellulose, NDF, ADF, and ADL compared to untreated control [28]. Moreover, they concluded that straw treated with *Pleurotus* spp. improved its nutritional value as livestock feed.

### 4.2. Gas and greenhouse gases

As previously mentioned, the significant interactions between ensiling duration and diet indicate that total gas production and GHG are diet and ensiling duration dependent at the same time. Gas production was reduced with the inclusion of SMS in the diets. The chemical composition of the substrates should be the main reason. In the present experiment, GP was positively correlated with OM, CP, EE, and NSC and negatively correlated with NDF, ADF, ADL, and hemicellulose. Diets containing high levels of SMS had more concentrations of these nutrients, which may partially explain these results.

The observed increases in GP with increasing the ensiling period may be related to changes in nutrient concentration during ensiling [34]. As the ensiling expanded, the concentration of NSC was increased in most of the diets, which offered an easy source of energy for ruminal microbes for their growth and other activities [35]. Moreover, increasing ensiling duration coincided with decreasing NDF, ADL, and hemicellulose concentrations. Such changes in the chemical composition may partially explain the GP values during different ensiling durations [36].

Diets containing SMS produced less GHG compared to the control diet, and as the proportion of SMS increased, the production of GHG decreased in most diets. From an environmental concern, there is potential for SMS to mitigate CH_4_ emissions. However, increasing ensiling duration increased the production of GHG, which is not desirable from an environmental perspective. Decreases in GHG production with SMS inclusion in the diets may be related to higher concentrations of OM and fermentable carbohydrates in these diets after the different ensiling durations. Additionally, Ngaowkakhiaw et al. [9] reported that the phenolic compounds in SMS can decrease CH_4_ production.

In the present study, negative correlations were observed (P < 0.01) between CH_4_ production and DM (r = −0.51), NDF (r = −0.66), ADF (r = −0.51), ADL (r = −0.44), hemicellulose (r = −0.44), *d*NDF (r = −0.27), *d*ADF (r = −0.20), *d*ADL (r = −0.44), and PF_24_ (r = −0.66), which may partially explain the observed lower CH_4_ production as these parameters were increased in diets containing SMS during the different ensiling durations. However, CH_4_ production was positively correlated with OM (r = 0.19), CP (r = 0.23), EE (r = 0.34), and NSC (r = 0.48), as well as GP (r = 0.78) and *d*DM (r = 0.52), and all these parameters were increased in diets containing SMS during the different ensiling durations. According to Boadi et al. [37], ruminal microbes produce more CH_4_ during structural carbohydrate fermentation than non-structural carbohydrates. Boadi and Wittenberg [38] stated that most of the dietary factors that reduce feed residence time will always result in low CH_4_ emissions. Factors such as high starch content and low fiber will favor C_3_ production, hence lower CH_4_ emissions [39]; however, a weak correlation between CH_4_ and C_3_ was noted in the present study. The relationship between CH_4_ production and C_3_ proportion was supported by McAllister and Newbold [40], who emphasized that diets that produce a low ratio of C_2_ to C_3_ will result in a decrease in CH_4_ production. In the present study, correlations between CH_4_ production with total and individual VFA were also weak.

Ngaowkakhiaw et al. [9] reported that replacing 50% of corn with SMS reduced gas production and CH_4_ emission due to increasing concentrations of tannins and phenolics in SMS, which can inhibit methanogenesis in the rumen [41]. In another study, Mahesh and Mohini [42] reported that low CH_4_ emissions have been recorded in diets containing cereal stover subjected to mushroom treatment. A study by Mahesh [43] revealed a linear reduction in CH_4_ from fungal-treated wheat straws, which had fewer fiber fractions than untreated straw. This might be due to a faster passage rate and shorter residence time associated with the digestion of less fibrous materials in the rumen. Sallam et al. [44] stated that this was probably due to the indirect effect of fiber digestion leading to shorter residency of feed particles in the rumen [45].

### 4.3. Nutrient disappearance, partitioning factor, and microbial mass

The absence of interactions between diet and ensiling duration on nutrient degradability, PF_24_, and MM shows that these variables are diet and duration independent. Increasing the proportion of SMS in the diet increased *d*DM in most diets, which could be linked to the fiber content and nutrient availability in the different diets. Lower *d*DM with SMS-containing diets suggests that lower proportions of SMS may enhance microbial efficiency in digesting the feed. Increasing SMS levels introduces more fibrous content, likely reducing digestibility. *d*DM had positive correlations (P < 0.01) with OM (r = 0.39), CP (r = 0.30), EE (r = 0.18), and NSC (r = 0.52) concentrations, indicating that other factors in the SMS could enhance *d*DM; however, *d*DM was negatively correlated with DM (r = −0.38), NDF (r = −0.70), ADF(r = −0.62), ADL(r = −0.44), hemicellulose (r = −0.39), and cellulose (r = −0.24). This aligns with a previous report by Li et al. [46], where an increase in CP content in SMS was associated with improved *d*DM.

Increasing the proportion of SMS in the diets increased *d*NDF, *d*ADF, and *d*ADL, which are good indicators of SMS's nutritive value as a feed. Higher *d*NDF and *d*ADF suggest that higher cellulose, hemicellulose, and lignin breakdown occurred, which is consistent with a previous study by Jung and Allen [47] who documented the impact of microbial activity on lignin degradation. In addition to the observed correlation between *d*NDF (negative) and *d*ADF (positive) with fiber fractions, it was suggested that SMS possibly provides a favorable environment for fiber-degrading microbes [48]. Additionally, the positive correlation between *d*NDF (r = 0.52) and MM can partially explain the results, since MM means more production of ruminal microbes during fermentation. Moreover, the observed increase in *d*ADL with SMS indicates the presence of specific microbial populations or their byproducts (e.g., extracellular enzymes) capable of lignin degradation or modification of lignin structure and facilitating its breakdown [47].

The results of increasing *d*NDF, *d*ADF, and *d*ADL with increasing ensiling durations confirm our assumptions that the fungi produced a complex array of enzymes and metabolites that improved the degradability of the fiber fractions. As mentioned before, *P. ostreatus* belongs to the basidiomycetes, which produce enzymes that are capable of degrading fiber fractions (de-lignification) [25]; de-lignification increases fiber solubility [25]. However, Olagunju et al. [6] observed a decrease in *d*NDF after treating corn stover with *P. ostreatus*, which could be due to the consumption of readily digestible substances present in the substrate by other microbes [49]. Increasing the proportion of SMS in the diets increased undegraded residuals and PF_24_ but decreased MM in most of the diets. The correlations between these parameters and the concentrations of different nutrients may partially explain the observed results.

Increasing ensiling durations increased *d*DM, PF_24_, and undegraded portion almost at all ensiling durations, indicating that more *d*DM was used to produce each gas unit. Additionally, PF_24_ was negatively correlated with OM (r = −0.23), CP (r = −0.27), EE (r = −0.31), NSC (r = −0.51), and *d*DM (r = −0.48) and positively correlated with NDF (r = 0.70), ADF (r = 0.43), ADL (r = 0.58), hemicellulose (r = 0.60), *d*ADF (r = 0.43), and *d*ADL (r = 0.41). These parameters were also increased or decreased with the inclusion of SMS in the diets. The positive correlations between MM and CP (r = 0.22) can also explain the observed results since protein is required to produce MM. Ngaowkakhiaw et al. [9] reported that replacing 50% of corn with SMS decreased *in vitro* true digestibility due to high cell wall content without affecting hemicellulose and cellulose digestibility.

### 4.4. Volatile fatty acids

The absence of interactions between diet and ensiling duration indicates that total and individual VFA are diet and duration independent. Diet type affected only the total VFA and butyrate proportion; however, it was expected that all individual VFA would differ between diets as previously noted by Kholif et al. [50], who reported that diet type affects total and individual VFA. Ensiling durations affected the concentration of total and proportions of individual VFA, which should be related to the chemical composition of the diets and the fermented products during the ensiling process. The CS90:SMS10 had the highest total VFA concentration and butyrate proportion, indicating that a possible synergy between CS and SMS occurred in that diet. However, increasing the SMS proportion in the diet decreased the concentration of total VFA and C_4_ proportion in most of the diets. Lower total VFA concentrations with increasing SMS inclusion in the diets indicate a suboptimal fermentation environment and suggest a potential inhibitory effect of SMS on ruminal fermentation, as previously reported by Kung et al. [48]. The correlation analysis showed that total VFA production was positively correlated with CP (r = 0.24) and MM (r = 0.28) and negatively with NDF (r = −0.28), ADF (r = −0.25), and *d*NDF (r = −0.18). All these parameters were affected by the inclusion of SMS in the diets.

Increasing the ensiling duration linearly increased C_2_ and C_3_, and decreased C_4_ and iso-C_4_ proportions in all diets, which may be due to the accumulation of C_2_ and C_3_ in the ensiled materials [34] or due to the carbohydrate composition and microbial population, as reported by Borreani et al. [51]. Moreover, reducing C_4_ production is desirable as its concentration is an indication of clostridial fermentation [52]. Olagunju et al. [6] reported that the treatment of corn stover with *P. ostreatus* increased total VFA and C_3_ and reduced C_2_ and C_2_:C_3_.

## 5. Conclusions

The use of *Pleurotus ostreatus*–based spent mushroom substrate in corn silage production shows promising results in altering fermentation characteristics, improving fiber digestibility, and modifying VFA profiles. These changes have significant implications for optimizing the nutritional quality of silage as a ruminant feed. The findings underscore the potential of spent mushroom substrate as a valuable additive in silage preparation, contributing to sustainable livestock feeding practices. Spent mushroom substrate can be included up to 50% to replace corn silage in the diets of beef and non-lactating dairy cows; however, extending the ensiling duration is not recommended. Further research is needed to explore the mechanisms behind these effects and optimize spent mushroom substrate proportions for specific outcomes in silage quality.

## Use of AI tools declaration

The authors declare they have not used Artificial Intelligence (AI) tools in the creation of this article.

## References

[1] Smith LC, Haddad L (2015). Reducing child undernutrition: Past Drivers and Priorities for the post-MDG era. World Dev.

[2] FAO (2017). The future of food and agriculture–Trends and challenges, Rome, Italy, FAO, United Nations, Rome.

[3] Tilman D, Clark M, Williams DR (2017). Future threats to biodiversity and pathways to their prevention. Nature.

[4] Mhlongo G, Mnisi CM, Mlambo V (2021). Cultivating oyster mushrooms on red grape pomace waste enhances potential nutritional value of the spent substrate for ruminants. PLoS One.

[5] Kousar A, Khan HA, Farid S (2024). Recent advances on environmentally sustainable valorization of spent mushroom substrate: A review. Biofuels Bioprod Biorefin.

[6] Olagunju LK, Isikhuemhen OS, Dele PA (2023). *Pleurotus ostreatus* can significantly improve the nutritive value of lignocellulosic crop residues. Agriculture (Switzerland).

[7] Mohd Hanafi FH, Rezania S, Mat Taib S (2018). Environmentally sustainable applications of agro-based spent mushroom substrate (SMS): An overview. J Mater Cycles Waste Manag.

[8] Kholif AE, Khattab HM, El-Shewy AA (2014). Nutrient digestibility, ruminal fermentation activities, serum parameters and milk production and composition of lactating goats fed diets containing rice straw treated with *Pleurotus ostreatus*. Asian-Australas J Anim Sci.

[9] Ngaowkakhiaw N, Kongmun P, Bungsrisawat P (2021). Effects of phytochemicals in spent mushroom substrate silage-based diets on methane mitigation by *in vitro* technique. Khon Kaen Agric J.

[10] Kholif AE, Gouda GA, Patra AK (2022). The sustainable mitigation of *in vitro* ruminal biogas emissions by ensiling date palm leaves and rice straw with lactic acid bacteria and *Pleurotus ostreatus* for cleaner livestock production. J Appl Microbiol.

[11] Pedroso A de F, Nussio LG, Paziani S de F (2005). Fermentation and epiphytic microflora dynamics in sugar cane silage. Sci Agric.

[12] Senger CCD, Mühlbach PRF, Sánchez LMB (2005). Chemical composition and ‘*in vitro*’ digestibility of maize silages with different maturities and packing densities. Ciência Rural.

[13] Alabi JO, Wuaku M, Anotaenwere CC (2024). A mixture of prebiotics, essential oil blends, and onion peel did not affect greenhouse gas emissions or nutrient degradability, but altered volatile fatty acids production in dairy cows using rumen simulation technique (RUSITEC). Fermentation.

[14] Ike KA, Adelusi OO, Alabi JO (2024). Effects of different essential oil blends and fumaric acid on *in vitro* fermentation, greenhouse gases, nutrient degradability, and total and molar proportions of volatile fatty acid production in a total mixed ration for dairy cattle. Agriculture.

[15] Hassan OGA, Hassaan NA, Kholif AE (2024). Influence of replacing soybean meal with nigella sativa seed meal on feed intake, digestibility, growth performance, blood metabolites, and antioxidant activity of growing lambs. Animals.

[16] Mousa GA, Kholif AE, Hassaan NA (2024). Effect of replacing cottonseed meal with fenugreek seed meal on feed intake, digestibility, growth, blood parameters and economics of fattening lambs. Small Rumin Res.

[17] Ike KA, Okedoyin DO, Alabi JO (2024). The combined effect of four nutraceutical-based feed additives on the rumen microbiome, methane gas emission, volatile fatty acids, and dry matter disappearance using an *in vitro* batch culture technique. Fermentation.

[18] Anele UY, Refat B, Swift ML (2014). Effects of bulk density, precision processing and processing index on *in vitro* ruminal fermentation of dry-rolled barley grain. Anim Feed Sci Technol.

[19] Anele UY, Südekum KH, Hummel J (2011). Chemical characterization, *in vitro* dry matter and ruminal crude protein degradability and microbial protein synthesis of some cowpea (*Vigna unguiculata* L. Walp) haulm varieties. Anim Feed Sci Technol.

[20] Blümmel M, Lebzien P (2001). Predicting ruminal microbial efficiencies of dairy rations by *in vitro* techniques. Livest Prod Sci.

[21] Blümmel M, Steingaβ H, Becker K (1997). The relationship between *in vitro* gas production, *in vitro* microbial biomass yield and ^15^N incorporation and its implications for the prediction of voluntary feed intake of roughages. Br J Nutr.

[22] Ruiz-Moreno M, Binversie E, Fessenden SW (2015). Mitigation of *in vitro* hydrogen sulfide production using bismuth subsalicylate with and without monensin in beef feedlot diets. J Anim Sci.

[23] Latimer George (2019). Official Methods of Analysis of AOAC International.

[24] Van Soest PJ, Robertson JB, Lewis BA (1991). Methods for dietary fiber, neutral detergent fiber, and nonstarch polysaccharides in relation to animal nutrition. J Dairy Sci.

[25] Ruggeri B, Sassi G (2003). Experimental sensitivity analysis of a trickle bed bioreactor for lignin peroxidases production by *P. chrysosporium*. Process Biochem.

[26] Khattab HM, Gado HM, Salem AZM (2013). Chemical composition and *in vitro* digestibility of *Pleurotus ostreatus* spent rice straw. Anim Nutr Feed Technol.

[27] Kwak WS, Kim YI, Seok JS (2009). Molasses and microbial inoculants improve fermentability and silage quality of cotton waste-based spent mushroom substrate. Bioresour Technol.

[28] Akinfemi A, Ogunwole OA (2012). Chemical composition and *in vitro* digestibility of rice straw treated with *Pleurotus ostreatus*, *Pleurotus pulmonarius* and *Pleurotus tuber-regium*. Slovak J Anim Sci.

[29] Adamovic M, Bocarov-Stancic A, Milenkovic I (2007). The quality of silage of corn grain and spent *P. ostreatus* mushroom substrate. Zb Matice Srp Prir Nauk.

[30] Kwak WS, Jung SH, Kim YI (2008). Broiler litter supplementation improves storage and feed-nutritional value of sawdust-based spent mushroom substrate. Bioresour Technol.

[31] Kim YI, Lee YH, Kim KH (2012). Effects of supplementing microbially-fermented spent mushroom substrates on growth performance and carcass characteristics of Hanwood steers (a field study). Asian-Australas J Anim Sci.

[32] Kim YI, Park JM, Lee YH (2014). Effect of by-product feed-based silage feeding on the performance, blood metabolites, and carcass characteristics of Hanwood steers (a field study). Asian-Australas J Anim Sci.

[33] Darwish GAMA, Bakr AA, Abdallah MMF (2012). Nutritional value upgrading of maize stalk by using *Pleurotus ostreatus* and *Saccharomyces cerevisiae* in solid state fermentation. Ann Agric Sci.

[34] Kholif AE, Gouda GA, Morsy TA (2024). The effects of replacement of berseem hay in total mixed rations with date palm leaves ensiled with malic or lactic acids at different levels on the nutritive value, ruminal *in vitro* biogas production and fermentation. Biomass Convers Biorefin.

[35] Kholif AE, Elghandour MMY, Salem AZM (2017). The effects of three total mixed rations with different concentrate to maize silage ratios and different levels of microalgae *Chlorella vulgaris* on *in vitro* total gas, methane and carbon dioxide production. J Agric Sci.

[36] Morsy TA, Gouda GAGA, Kholif AE (2022). *In vitro* fermentation and production of methane and carbon dioxide from rations containing *Moringa oleifera* leave silage as a replacement of soybean meal: *In vitro* assessment. Environ Sci Pollut Res.

[37] Boadi D, Benchaar C, Chiquette J (2004). Mitigation strategies to reduce enteric methane emissions from dairy cows: Update review. Can J Anim Sci.

[38] Boadi DA, Wittenberg KM (2002). Methane production from dairy and beef heifers fed forages differing in nutrient density using the sulphur hexafluoride (SF6) tracer gas technique. Can J Anim Sci.

[39] Sallam SMA, Rady AMS, Attia MFA (2024). Different maize silage cultivars with or without urea as a feed for ruminant: Chemical composition and *in vitro* fer-mentation and nutrient degradability. Chil J Agric Anim Sci.

[40] McAllister TA, Newbold CJ (2008). Redirecting rumen fermentation to reduce methanogenesis. Aust J Exp Agric.

[41] Aslam S (2013). Organic management of root knot nematodes in tomato with spent mushroom compost. Sarhad J Agric.

[42] Mahesh MS, Madhu M (2013). Biological treatment of crop residues for ruminant feeding: A review. Afr J Biotechnol.

[43] Mahesh MS (2012). Fungal bioremediation of wheat straw to improve the nutritive value and its effect on methane production in ruminants. MVSc thesis submitted to National Dairy Research institute (Deemed University), Karnal, Haryana, India, 2012.

[44] Sallam SMA, Nasser MEA, El-Waziry AM (2007). Use of an *in vitro* rumen gas production technique to evaluate some ruminant feedstuffs. J Appl Sci Res.

[45] Kholif AE, Gouda GA, Morsy TA (2022). Dietary date palm leaves ensiled with fibrolytic enzymes decreased methane production, and improved feed degradability and fermentation kinetics in a ruminal *in vitro* system. Waste Biomass Valorization.

[46] Li X, Pang Y, Zhang R (2001). Compositional changes of cottonseed hull substrate during *P. ostreatus* growth and the effects on the feeding value of the spent substrate. Bioresour Technol.

[47] Jung HG, Allen MS (1995). Characteristics of plant cell walls affecting intake and digestibility of forages by ruminants. J Anim Sci.

[48] Kung L, Shaver RD, Grant RJ (2018). Silage review: Interpretation of chemical, microbial, and organoleptic components of silages. J Dairy Sci.

[49] Zadrazil F (1997). Changes in *in vitro* digestibility of wheat straw during fungal growth and after harvest of oyster mushrooms (*Pleurotus* spp.) on laboratory and industrial scale. J Appl Anim Res.

[50] Kholif AE, Gouda GA, Morsy TA (2023). Associative effects between *Chlorella vulgaris* microalgae and *Moringa oleifera* leaf silage used at different levels decreased *in vitro* ruminal greenhouse gas production and altered ruminal fermentation. Environ Sci Pollut Res.

[51] Borreani G, Tabacco E, Schmidt RJJ (2018). Silage review: Factors affecting dry matter and quality losses in silages. J Dairy Sci.

[52] Bedford A, Gong J (2018). Implications of butyrate and its derivatives for gut health and animal production. Anim Nutr.

